# Risk of intussusception after monovalent rotavirus vaccine (Rotavac) in Indian infants: A self-controlled case series analysis

**DOI:** 10.1016/j.vaccine.2020.09.019

**Published:** 2021-01-03

**Authors:** The INCLEN Intussusception Surveillance Network Study Group, Manoja Kumar Das, Narendra Kumar Arora, Ramesh Poluru, Jacqueline E. Tate, Bini Gupta, Apoorva Sharan, Mahesh K. Aggarwal, Pradeep Haldar, Umesh D Parashar, Patrick L.F. Zuber, Jan Bonhoeffer, Arindam Ray, Ashish Wakhlu, Bhadresh R. Vyas, Javeed Iqbal Bhat, Jayanta K. Goswami, John Mathai, Kameswari K., Lalit Bharadia, Lalit Sankhe, Ajayakumar M.K., Neelam Mohan, Pradeep K. Jena, Rachita Sarangi, Rashmi Shad, Sanjib K. Debbarma, Shyamala J., Simmi K. Ratan, Suman Sarkar, Vijayendra Kumar, Christine G. Maure, Anand P. Dubey, Atul Gupta, Cenita J. Sam, Gowhar Nazir Mufti, Harsh Trivedi, Jimmy Shad, Kaushik Lahiri, Krishnaswamy R, Meera Luthra, Narendra Behera, Padmalatha P, Rajamani G., Rakesh Kumar, Ruchirendu Sarkar, Santosh Kumar A., Subrat Kumar Sahoo, Sunil K. Ghosh, Sushant Mane, Arun Dash, Bashir Ahmad Charoo, Bikasha Bihary Tripathy, Rajendra Prasad G., Harish Kumar S, Jothilakshmi K, Nihar Ranjan Sarkar, Pavai Arunachalam, Satya Sundar G. Mohapatra, Saurabh Garge

**Affiliations:** a The INCLEN Trust International, F1/5, Okhla Industrial Area, Phase 1, New Delhi 110020, India; b National Center for Immunization and Respiratory Diseases, Centers for Disease Control and Prevention Atlanta, GA, USA; c Deputy Commissioner-Immunization, Ministry of Health & Family Welfare, Government of India, New Delhi, India; d Global Vaccine Safety and Vigilance Team, World Health Organization, Geneva, Switzerland; e Brighton Collaboration Foundation, Infectious Diseases and Vaccines, University Children’s Hospital, Basel, Switzerland; f Bill and Melinda Gates Foundation, India Country Office, New Delhi, India; g Department of Paediatric Surgery, King George’s Medical University, Lucknow, Uttar Pradesh, India; h Department of Paediatrics, MP Shah Government Medical College, Jamnagar, Gujarat, India; i Department of Paediatrics, Sher-I-Kashmir Institute of Medical Sciences, Srinagar, Jammu & Kashmir, India; j Department of Paediatric Surgery, Gauhati Medical College, Guwahati, Assam, India; k Department of Paediatrics, PSG Institute of Medical Sciences, Coimbatore, Tamil Nadu, India; l Department of Paediatric Surgery, Andhra Medical College, Vishakhapatnam, Andhra Pradesh, India; m Consultant Paediatric Gastroenterologist, Fortis Escorts Hospital, Jaipur, Rajasthan, India; n Department of Community Medicine, Grant Medical College & JJ Hospital, Mumbai, Maharashtra, India; o Department of Paediatric Surgery, Government Medical College & SAT Hospital, Thiruvananthapuram, Kerala, India; p Consultant Paediatrics Gastroenterology, Medanta—The Medicity, Gurgaon, Haryana, India; q Department of Paediatric Surgery, SCB Medical College, Cuttack, Odisha, India; r Department of Paediatrics, IMS & SUM Medical College & Hospital, Bhubaneswar, Odisha, India; s Choithram Hospital and Research Centre, Indore, Madhya Pradesh, India; t Department of Paediatrics, Agartala Government Medical College, Agartala, Tripura, India; u Consultant Pediatrics, Apollo Hospitals, Chennai, Tamil Nadu, India; v Department of Paediatric Surgery, Maulana Azad Medical College, Delhi, India; w Department of Paediatrics, Institute of Post Graduate Medical Education and Research, Kolkata, West Bengal, India; x Department of Paediatric Surgery, Indira Gandhi Institute of Medical Sciences, Patna, Bihar, India; y Global Vaccine Safety and Vigilance Team, World Health Organization, Geneva, Switzerland; z Department of Paediatrics, Maulana Azad Medical College, Delhi, India; aa Consultant Pediatric Surgery, Fortis Escorts Hospital, Jaipur, Rajasthan, India; ab Department of Paediatric Surgery, PSG Institute of Medical Sciences, Coimbatore, Tamil Nadu, India; ac Department of Paediatric Surgery, Sher-I-Kashmir Institute of Medical Sciences, Srinagar, Jammu & Kashmir, India; ad Department of Paediatric Surgery, MP Shah Government Medical College, Jamnagar, Gujarat, India; ae Chief Medical Officer, Masonic Children's Hospital, Coimbatore, Tamil Nadu, India; af Consultant Paediatric Surgery, Medanta- The Medicity, Gurgaon, Haryana, India; ag Department of Pediatrics, MKCG Medical College, Berhampur, Odisha, India; ah Department of Paediatrics, Andhra Medical College, Vishakhapatnam, Andhra Pradesh, India; ai Department of Paediatric Surgery, Coimbatore Medical College, Coimbatore, Tamil Nadu, India; aj Department of Paediatrics, Indira Gandhi Institute of Medical Sciences, Patna, Bihar, India; ak Department of Paediatric Surgery, Institute of Post Graduate Medical Education and Research, Kolkata, West Bengal, India; al Department of Paediatrics, Government Medical College & SAT Hospital, Thiruvananthapuram, Kerala, India; am Department of Paediatric Surgery, IMS & SUM Medical College & Hospital, Bhubaneswar, Odisha, India; an Department of Pediatric Surgery, Agartala Government Medical College, Agartala, Tripura, India; ao Department of Paediatrics, Grant Medical College & JJ Hospital, Mumbai, Maharashtra, India; ap Department of Pediatric Surgery, MKCG Medical College, Berhampur, Odisha; aq Department of Paediatrics, Sher-I-Kashmir Institute of Medical Sciences, Srinagar, Jammu & Kashmir, India; ar Department of Paediatric Surgery, IMS & SUM Medical College & Hospital, Bhubaneswar, Odisha, India; as Department of Radiology, Institute of Post Graduate Medical Education and Research, Kolkata, West Bengal, India; at Department of Radiology, IMS & SUM Medical College & Hospital, Bhubaneswar, Odisha, India

**Keywords:** Intussusception, Rotavirus vaccine, Vaccine safety, Self-controlled case-series, Rotavac, Infant, India, CRF, Case record form, CAC, Case Adjudication Committee, CI, Confidence interval, ICD, International Classification of Diseases, IQR, Interquartile range, LMIC, Low and middle income countries, MIC, Middle income countries, NIP, National immunization programmes, SCCS, Self-controlled case series, RI, Relative incidence, RR, Relative risk, RV1, Monovalent rotavirus vaccine (Rotarix™), RV5, Pentavalent rotavirus vaccine (Rotateq™), RVV, Rotavirus vaccine, TAG, Technical Advisory Group, WHO, World Health Organization

## Abstract

**Background:**

An association between rotavirus vaccination and intussusception has been documented in post-licensure studies in some countries. We evaluated the risk of intussusception associated with monovalent rotavirus vaccine (Rotavac) administered at 6, 10 and 14 weeks of age in India.

**Methods:**

Active prospective surveillance for intussusception was conducted at 22 hospitals across 16 states from April 2016 through September 2017. Data on demography, clinical features and vaccination were documented. Age-adjusted relative incidence for 1–7, 8–21, and 1–21 days after rotavirus vaccination in children aged 28–364 days at intussusception onset was estimated using the self-controlled case-series (SCCS) method. Only Brighton Collaboration level 1 cases were included.

**Results:**

Out of 670 children aged 2–23 months with intussusception, 311 (46.4%) children were aged 28–364 days with confirmed vaccination status. Out of these, 52 intussusception cases with confirmed receipt of RVV were included in the SCCS analysis. No intussusception case was observed within 21 days of dose 1. Only one case occurred during 8–21 days after the dose 2. Post-dose 3, two cases in 1–7 days and 7 cases during 8–21 days period were observed. There was no increased risk of intussusception during 1–7 days after the doses 1 and 2 (zero cases observed) or dose 3 (relative incidence [RI], 1.71 [95% confidence interval {CI} 0.0–5.11]). Similarly, no increased risk during 8–21 days after the dose 1 (zero cases observed), dose 2 (RI, 0.71 [95% CI, 0.0–3.28]) or dose 3 (RI, 2.52 [95% CI, 0.78–5.61]). The results were similar for 1–21 day periods after the doses separately or pooled.

**Conclusions:**

The risk of intussusception during the first 21 days after any dose of rotavirus vaccine (Rotavac) was not higher among the Indian infants than the background risk, based on limited SCCS analysis of 52 children.

## 1 Introduction

To prevent diarrhoea related deaths, 107 countries (103 nationally and 4 sub-nationally) have introduced rotavirus vaccine (RVV) into their national immunization program (NIP) as of April 2020 [1]. The impact of RVV on the illness episodes, hospitalisations and deaths due to rotavirus and all-cause diarrhoeas has been documented from post-licensure studies from various countries [2], [3], [4], [5].

Following the increased risk of intussusception documented with the first licensed rotavirus vaccine (RotaShield™, Wyeth-Lederle Laboratories) [6] and its withdrawal, all the clinical trials of RVV captured intussusception as an adverse event. No increased risk of intussusception was observed in large scale multicountry clinical trials of two RVVs (Rotarix™, RV1; GlaxoSmithKline Biologicals and RotaTeq™, RV5; Merck & Co, Inc) [7], [8]. However, several postlicensure studies have identified some increased risk of intussusception during 1–7 days after the first (relative risk, RR: 5.3–9.9) and second (RR: 1.3–2.8) doses of these two RVVs in different countries (Mexico, Brazil, Australia, the United Kingdom, and the United States) [9], [10], [11], [13], [14]. Notably, the impact of RVV on morbidity and mortality outweigh the risk of intussusception and associated mortality [15].

Intussusception incidence varies widely across the countries [16]. In India, information on incidence of intussusception is limited and it varies from 17.7 (Delhi, North India) to 254 (Vellore, South India) cases per 100,000 child-years [17]. Although the exact causes of the variation in intussusception rate remain unknown, ethnicity, dietary pattern, breastfeeding practices, gut microbial environment, and vertical transmission of rotavirus antibodies have been proposed as the possible risk factors [16].

Out of the four RVVs licensed in India, two Indian (Rotavac™, RV1-116E; Bharat Biotech and Rotasill™, RV5; Serum. Institute of India) have undergone efficacy trials in India and remaining two (Rotarix™ and RotaTeq™) were licensed based on evidence from other countries. Clinical trials of the Indian-manufactured rotavirus vaccines enrolled 6799–7500 infants each and had inadequate sample size for documenting the risk of intussusception [18], [19]. Under the NIP, India introduced Rotavac™ in March 2016 and Rotasill™ in April 2018 in different states in a phased manner [20]. All four licensed vaccines are being used in private sector. In view of the variable risks of intussusception after RVV doses across the countries, it is necessary to generate evidence from the Indian context to sustain confidence of the professionals and public on the vaccine and program.

As part of the vaccine safety surveillance linked to RVV introduction in India, a nationally representative sentinel surveillance network was established. We used the self-controlled case series (SCCS) method to evaluate the risk of intussusception following Rotavac vaccination in Indian infants.

## 2 Methods

### 2.1 Study area and participating hospitals

This active prospective surveillance was conducted during April 2016 to September 2017 at 23 tertiary care hospitals across 20 cities and 17 states in India representing different regions. The list and location of these hospitals are given in Supplementary Figure SF1. These hospitals were selected through a systematic process and the study protocol has been published earlier [21].

### 2.2 Case recruitment and data collection

Children aged >1 month and <24 months admitted to these hospitals were screened to identify the suspected cases (any of these diagnoses: intussusception, intestinal obstruction- acute or subacute, acute abdomen, pain abdomen, abdominal distension, and blood in stool with vomiting). These suspected cases tracked for final diagnosis and confirmed intussusception cases meeting the Brighton collaboration level 1 criteria for diagnostic certainty were recruited. The cases were recruited irrespective of the immunization exposure and data availability. For the recruited cases, data on clinical features, investigations, treatment, outcome, and socio-demography were obtained from the hospital records and parent interview. Immunization data were collected from the immunization cards and a copy of which was obtained, whenever possible. An independent Case Adjudication Committee (CAC), with a paediatrician, paediatric surgeon, and radiologist as members, reviewed the documents to assign the diagnostic certainty levels, according to Brighton Collaboration criteria [22]. Independently the suspected and intussusception cases were verified from the medical records using diagnoses and/or International Classification of Diseases (ICD) codes (ICD-9/10, codes listed in Supplementary Table ST1), to identify any missed cases.

### 2.3 Statistical analysis

We used the SCCS method to estimate the age-adjusted relative incidence of intussusception for the periods 1–7, 8–21 and 1–21 days after each dose of the RVV among the children aged 28–364 days at onset of symptoms (primary analysis). In the SCCS method, each case acts as its own control and the frequency of the outcome (intussusception) that occurred in the exposure periods after vaccination is compared with the frequency in the unexposed period [23], [24]. As no external controls are used, this method automatically controls for time-invariant confounding [25]. The SCCS has been used in several studies investigating the risk of intussusception with RVV vaccine [9], [10], [11], [14], [23], [26], [27], [28], [29] with the pseudo-likelihood method [30]. We limited the analysis to the first occurrences of intussusception that met the level-1 Brighton collaboration criteria and children aged 28–364 days at the onset of symptoms, considering the minimum and maximum ages at which RVV could be given in India. Days 22 days after the RVV dose till 364 days of age were considered as the control period. Onset of first compatible symptom was considered the date of intussusception onset. Cases with confirmed vaccine exposure information were included in analysis and those without vaccination card were excluded. The relative incidence (RI) for each case was calculated comparing the incidence of intussusception within the risk window with the incidence in all other observation widows using conditional Poisson regression. Because the background incidence of intussusception varies substantially by age in the first year of life, age was controlled using 14-day intervals in the model and confidence intervals (CIs) were derived by bootstrapping with 1000 iterations and all cases were included. Sensitivity analysis was done considering the admission date as the date of intussusception onset. *P* values < 0.05 were considered to be statistically significant and all reported *P* values are two-sided. Statistical analysis was performed using STATA version 15.0 (StataCorp LLC, Texas, USA).

### 2.4 Sample size

To detect a relative incidence of 1.75 within 1–7 days of after any of the 3 RVV doses with 80% power and 95% confidence level, the sample size needed was 220 using the signed root likelihood ratio [31].

### 2.5 Ethical issues

Informed written consent was obtained for all the eligible cases from parent or legal guardian before recruitment and data collection. Confidentiality in data handling was maintained. The study protocol was reviewed and approved by all the institute ethics committees of participating institutes.

## 3 Results

**Fig. 1:**
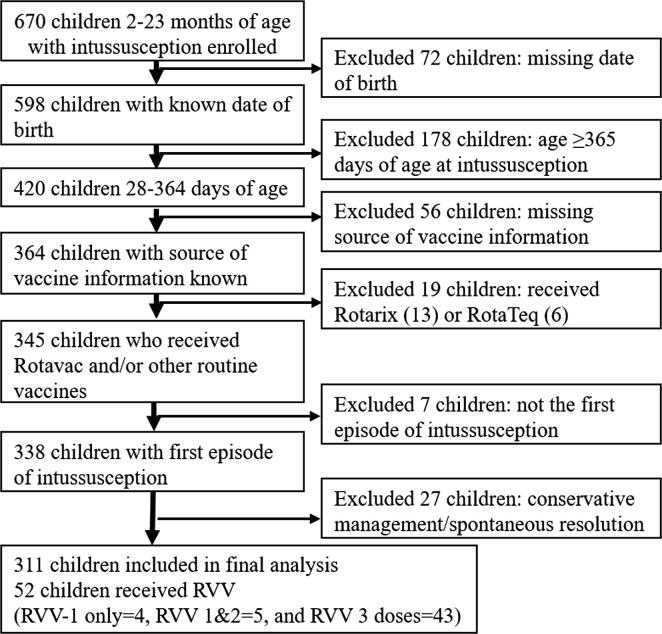
Flow of case selection for the self-controlled case series analysis. *Note: RVV: Rotavirus vaccine.*

A total of 670 children aged >1 month and <24 months with intussusception were recruited at these study sites. Among these, 420 (62.7%) children were aged 28–364 days. Out of the infants, 86.4% (364/420) had vaccine exposure information and 80.5% (338/420) had the first episode of intussusception. The 27 (4%) children who resolved with conservative management (i.e. no radiologic or surgical intervention) were excluded. The 19 (2.8%) children who received either Rotarix™ or Rotateq™ were also excluded. Thus data for 311 children (52 with and 259 without RVV) were analysed. The Fig. 1 shows the selection of cases for SCCS analysis.

**Fig. 2:**
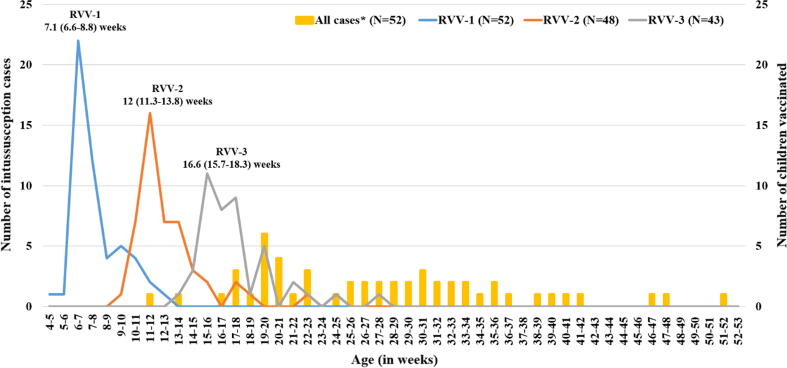
The age at rotavirus vaccination and occurrence of intussusception during the first year. *Note: The ages at rotavirus vaccination given in weeks as median with interquartile range (IQR); RVV-1: Rotavirus vaccine first dose; RVV-2: Rotavirus vaccine second dose; RVV-3: Rotavirus vaccine third dose.*

There were no significant differences in the age at onset of symptoms, gender and interval between the onset and hospitalisation between the cases included in analysis and excluded (Supplementary Table ST2). The median age of cases was 28 weeks (interquartile range [IQR], 22–35 weeks) and 68.2% were boys (Supplementary Table ST2). Vomiting (78.8%), blood in stool (71.1%), excessive crying (62.4%) and abdominal pain (49.8%) were the commonest presenting features (Supplementary Table ST3). The median interval between onset of symptoms and hospitalization was 3-days (IQR, 2–6 days). Overall, RVV was received by 52 (16.7%) cases including three doses by 43, two doses by 5 and one dose by 4 cases. Among the 53 cases recruited from the five sites from states in Phase-1 RVV national introduction, 34 (64.1%) received RVV-1. Out of the 258 children recruited from the 15 sites in the states without RVV in the NIP, 18 (7%) received RVV-1. The median ages at intussusception were comparable for the children who received RVV (26.5 weeks; IQR 19.5–32.5 weeks) and who did not receive (28 weeks; IQR 22–36 weeks) (Supplementary Table ST5). The median ages of vaccination in children with and without RVV were comparable; dose 1 (with RVV: median age 7.1 weeks, IQR 6.6–8.8 weeks and without RVV: median age 7 weeks, IQR 6.6–8.4 weeks), dose 2 (with RVV: median age 12 weeks, IQR 11.3–13.8 weeks and without RVV: median age 12.3 weeks, IQR 11.3–14.1 weeks) and dose 3 (with RVV: median age 16.6 weeks, IQR 15.7–18.3 weeks and without RVV: median age 17.1 weeks, IQR 16–19.1 weeks). The Fig. 2 shows the ages at vaccination for the children with RVV and the ages of vaccination for children without RVV is given as Supplementary Figure SF2. There was no significant difference in the characteristics between the children who did or didn’t receive RVV (Supplementary Table ST5).

**Fig. 3:**
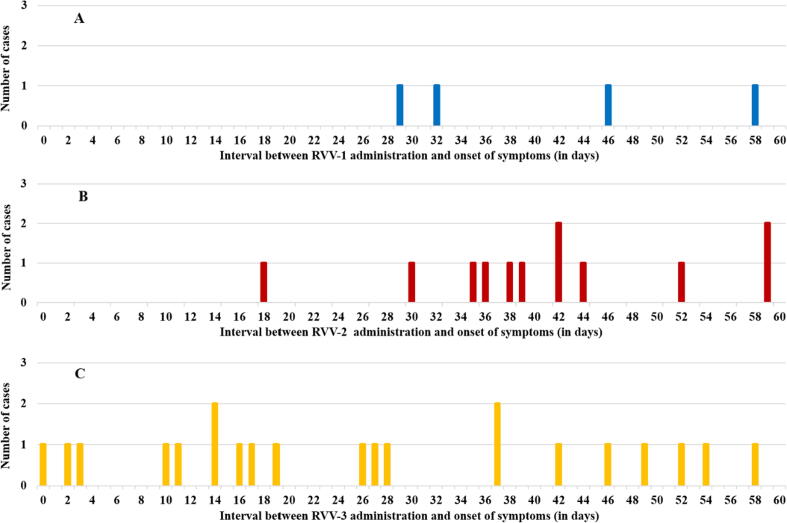
The number of intussusception cases in the first 60 days after rotavirus vaccine dose 1 (3A), dose 2 (3B) and dose 3 (3C). *Note: RVV-1: Rotavirus vaccine first dose; RVV-2: Rotavirus vaccine second dose; RVV-3: Rotavirus vaccine third dose.*

After the first RVV dose, no case of intussusception was observed during the 1–7 days and 8–21 days risk periods (Fig. 3A). After the second RVV dose, no case of intussusception during the 1–7 days and one case during 8–21 days risk periods were observed (Fig. 3B). After the third RVV dose, two cases of intussusception during the 1–7 days and seven cases during 8–21 days risk periods were observed (Fig. 3C). One case occurred on day zero after RVV dose 3.

Table 1 shows the RI estimates using the date of symptom onset (primary analysis) and date of admission (sensitivity analysis) for intussusception in different risk periods (1–7 days or 8–21 days or 1–21 days) after the RVV doses compared to the background risk. There was no increased risk of intussusception during 1–7 days after the first and second doses (no cases observed) or third dose (relative incidence, RI, 1.03; 95% CI 0–5.11) of RVV. Similarly, no increased risk of intussusception was observed during 8–21 risk period after the first dose (no case observed), second dose (RI 0.71; 95% CI 0–3.28) and third dose (RI 2.52; 95% CI 0.78–5.61) of RVV. There was no increased risk of intussusception observed for any RVV dose at pooled level for 1–7, 8–21 and 1–21 days risk periods.

**Table 1:** Relative incidence of intussusception in the risk periods after the rotavirus vaccine in Indian infants.

Dose	Risk period	Primary analysis*				Sensitivity analysis**			
		IS cases (n)	Relative incidence (RI with 95% CI)@			IS cases (n)	Relative incidence (RI with 95% CI)@		
	1–7 days	0	0.00	0.00	0.00	0	0.00	0.00	0.00
Dose-1	8–21 days	0	0.00	0.00	0.00	0	0.00	0.00	0.00
	1–21 days	0	0.00	0.00	0.00	0	0.00	0.00	0.00
Dose-2	1–7 days	0	0.00	0.00	0.00	0	0.00	0.00	0.00
	8–21 days	1	0.71	0.00	3.28	1	0.74	0.00	3.66
	1–21 days	1	0.50	0.00	2.37	1	0.52	0.00	2.59
Dose-3	1–7 days	2	1.71	0.00	5.11	3	2.56	0.00	6.92
	8–21 days	7	2.52	0.78	5.61	6	2.11	0.61	4.72
	1–21 days	9	2.23	0.81	4.58	9	2.20	0.88	4.41
All 3-doses	1–7 days	2	1.03	0.00	3.14	3	1.61	0.00	4.43
	8–21 days	8	1.65	0.61	3.49	7	1.46	0.51	3.15
	1–21 days	10	1.48	0.67	3.02	10	1.50	0.70	2.92

Notes: Among children aged 28–364 days; Risk period is number of days prior to the reference date; IS: Intussusception; RI: Relative Incidence.

* Using the symptom onset date for calculating the risk periods.

** Using the admission date as onset of intussusception for calculating the risk periods.

@ 95% confidence intervals bootstrapped with 1000 iterations.

## 4 Discussion

This study was conducted at 20 sites as part of the vaccine safety surveillance linked to rotavirus vaccine introduction. Out of the 20 sites, five sites were in the states where the RVV was introduced in Phase-1 of the national introduction. According to the WHO and UNICEF estimates of immunization coverage using the administrative report for 2017, the RVV (Rotavac™) coverage in these Phase-1 states was 69% [32]. The RVV coverage (64.1%) among the study subjects from the study sites in Phase-1 states was comparable to the administrative coverage data. Out of the 258 children recruited from other 15 sites (from the states without RVV in the NIP), 18 (7%) children received RVV. We did not observe an increased risk of intussusception after any of the doses of the monovalent 116E RVV (Rotavac™) in Indian infants. No clustering of the intussusception cases in either 1–7 days or 8–21 days or 1–21 days risk windows after any RVV doses was observed. However, the study was under powered due to the low number of intussusception cases having received RVV (n = 52).

Our results are comparable to the reports of intussusception after RV1 (Rotarix™) vaccination in several African countries (Ethiopia, Ghana, Kenya, Malawi, Tanzania, Zambia, Zimbabwe and South Africa), where no increased risk of intussusception after any dose of RV1 were observed [23], [28]. However, increased risks of intussusception were documented in the 1–7 days following dose 2 of RV1 in Brazil (IR 2.6; 95% CI 1.3–5.2; OR 1.9; 95% CI 1.1–3.4) and after dose 2 (IR 5.3; 95% CI 3.0–9.3 and OR 5.8; 95% CI 2.6–13) and dose 1 (IR 6.49; 95% CI 4.17–10.09) in Mexico [9], [10]. Similarly, increased risks of intussusception were documented in high income countries, United States (first dose, RR 7–8.8; second dose, RR 1.8–8.1), United Kingdom (first dose, RR 13.8; second dose, RR 2.2), and Australia (first dose, RR 6.8; second dose, RR 2.8) in the 1–7 day period with RV1 vaccine [11], [12], [13], [14]. Increased risks of intussusception were documented in high income countries in United States (first dose, RR 2.6–9.1; second dose, RR 1.8; third dose, RR 1.2–2.2), Finland (first dose, IRR 2) and Australia (first dose, RR 9.9; second dose, RR 2.8) in the 1–7 day period with RV5 vaccine (Rotateq™) [11], [12], [13], [29]. In Singapore (first dose, RR 8.3; second dose, RR 3.0)and Spain (first dose, RR 4.7; second dose, RR 1.6) also the intussusception risk was higher where both RV1 and RV5 were used [26], [27]. The RV1 doses are administered at 2 and 3 or 4 months schedule. The RV5 doses are administered at 2, 4 and 5 or 6 months schedule.

Although the exact reasons of increased risk of intussusception with RVV in some countries but not in others are not clear, some possible factors like age at vaccination, coadministration of oral polio vaccine (OPV), gut replication of RVV virus, intestinal microbiota environment, breastfeeding and dietary patterns, maternal antibody transfer and ethnicity may be considered [33]. In this study, the median age of the intussusception cases is 28 weeks (IQR, 22–35 weeks), which is comparable to the reports for Indian infants without RVV exposure [34], [35], [36]. In this study no case was observed before 6 weeks, one case before 10 weeks and 11 cases before 14 weeks of ages. This observation is similar to that from African countries [23], [28]. The median or peak ages of intussusception in children from the countries with increased risk were also comparable to the Indian and African children. It was interesting to note that the countries following 6 and 10 weeks (African countries) and 6, 10 and 14 weeks (India) schedules have no or lower risk of intussusception [23], [28]. But the countries following 2 and 3 months or 2 and 4 months schedules for RV1 and 2, 3 and 5 months or 2, 4 and 6 months schedules for RV5 had higher risk of intussusception [9], [10], [11], [12], [13], [14], [26], [27], [28], [29]. Risk of intussusception was highest in 1–7 days after the first dose or either RV1 or RV5. In this study, we observed one case on day-zero of RVV dose-3. Apart from the schedule, while no increased risk of intussusception was observed in the low and middle income countries (LMICs) from Africa and India, the middle income countries (MICs) from Latin America and developed countries from North America, Europe and Australia had increased risks of intussusception. So, the earlier age of administration of first RVV dose may have lower risk of intussusception, when the background occurrence is low. The RVV administered at 10–14 weeks schedule was observed to have higher seroconverion compared to 6–10 weeks schedule [37]. In India and the African countries OPV is coadministered with RVV. In this study, 47 children received RVV and OPV simultaneously. The co-administration of RVV and OPV was observed to have lower rotavirus seroconversion in South Africa, Bangladesh and Chile infants [37], [38], [39]. The lower seroconversion may be due to sub-optimal RVV virus replication in the intestine.

Lower efficacy (49.7–64.5%) in clinical trials and effectiveness (18–69%) of the RVVs have been observed in LMICs from Africa, Latin America and Asia compared to the middle and high income countries [40], [5]. The lower immune responses to the RVVs may be due to lower replication of the RVV viruses in the intestine of infants in LMICs, which is reflected in lower fecal shedding [33]. The lower replication of RVV viruses may be dependent on the intestinal microbiota and other competing microbes of the infants in these LMICs [33], [41]. The intestinal replication of virus may be influenced by the breastfeeding pattern, immunoglobulin and non-specific antibodies content in the breastmilk, dietary pattern, environmental sanitation and nutritional status of the infants [33], [41]. Apart from these the maternal infection with rotavirus and vertical transfer to infants may also affect the vaccine virus replication, and thereby the immunogenicity and risk of intussusception [33], [41]. The variation in the intussusception rates across the different regions globally may also be due to genetic and/or ethnic risk factors [16], [41]. With these possible factors influencing the intussusception risk after RVV, the subnational variations of the risks in India should also be explored, considering the wide variation in the dietary, sociocultural practices, sanitation and environmental risk factors.

Our study had some limitations. First, the number of children who received RVV was small and is underpowered to observe significant risk of intussusception. However, we did not observe any case of intussusception during 1–7 days after the first and second doses of RVV, which was reassuring. There were two cases of intussusception (ages 18.2 and 25.3 weeks) during 1–7 days after third dose of RVV, which overlaps with the age of natural occurrence of intussusception in infants, even without RVV. The observations are similar to a recent report from India on intussusception after Rotavac, where out of 104 intussusception cases, none occurred during 1–7 days after RVV1 and RVV2 while one case occurred during 1–7 days after RVV3. The relative incidences of intussusception during 1–21 days after RVV1 and RVV2 (RI: 1.56; 95% CI: 0.0–5.28) and any RVV dose (RI: 1.51; 95% CI: 0.58–3.23). The findings from our study are comparable to observations from this report [41], [42]. Second, only cases from selected hospitals were included and no definite catchment area or population base could be ascertained to derive the incidence rate. Third, all the children with intussusception were recruited at the sites irrespective of the immunization exposure status. Several of the infants were from outside the city and their parents were not carrying the vaccine cards with them. We surveillance team tried to collect the vaccination information to their best. The vaccine exposure information was available for 78.4% of infants, which corroborates with the immunization coverage status of infants in India and there was no significant difference in the age distribution between the infants who were included in analysis and those who were not. The key parameters for the vaccinated and unvaccinated infants appeared similar. We had study sites across 17 states of India and had mix of public and private hospitals and we believe that the findings are generalizable to other areas of India.

In conclusion, this SCCS analysis did not observe any increased risk of intussusception in the 1–7 and 8–21 days periods after the any dose of the oral monovalent 116E RVV (Rotavac™) among Indian infants compared to the background risk. This observation is similar to the risks observed among African infants following oral RV1 administration from post-licensure studies [23], [28]. Although no increased risk of intussusception with the RVV in Indian infants is encouraging, continued documentation over longer period and sub-national risk assessment should also be considered. Such evidence will also be useful for other counties in the region and globally considering use of these RVVs for preventing diarrhoea morbidity and deaths.

## 5 Consent for publication

Not applicable.

## 6 Availability of data and materials

All data is available with the investigators and can be provided by the corresponding author upon reasonable request.

## 7 Disclosure statement

None. There is no financial interest or benefit for the authors arisen from this project or its direct application.

## 8 Funding xxx

This project was supported by the 10.13039/100000865Bill and Melinda Gates Foundation, USA to The INCLEN Trust International (grant number OPP1116433). The funder or its representative had no role in the design of the study and collection, analysis, and interpretation of data and writing the manuscript.

## 9 Authors’ contributions xxx

MKD and NKA conceptualised the framework for the study protocol, training, data analysis and interpretation. All TAG members provided input for finalisation of the study protocol and provided quality assurance oversight. All site investigators supervised the data collection at respective study site institutes. BG and AS coordinated the data collection and collation. MKD, RR, JET and BG analysed the data. MKD and RR wrote the first draft of the manuscript. JET and UP reviewed and revised the manuscript. All authors reviewed, provided critical input and approved the final version.

## 10 Disclaimer

The content represents the views of the authors alone and do not necessarily represent the official positions of their organizations, World Health Organization or Ministry of Health and Family Welfare, Government of India or the US Centers for Disease Control and Prevention or Bill and Melinda Gates Foundation.

## CRediT authorship contribution statement

**Manoja Kumar Das and Narendra Kumar Arora:** Study conceptualisation, study design, protocol development, training, data analysis, interpretation, manuscript preparation.

**Bini Gupta and Apoorva Sharan:** Study coordination, monitoring, data cleaning, data analysis.

**Ramesh Poluru, Jacqueline E. Tate and Umesh D. Parashar:** Protocol development, data analysis and manuscript preparation.

**Mahesh K. Aggarwal, Pradeep Haldar, Patrick L F Zuber, Jan Bonhoeffer, Arindam Ray and Christine G. Maure:** Protocol development, quality assurance and monitoring.

**Ashish Wakhlu, Bhadresh R Vyas, Javeed Iqbal Bhat, Jayanta K. Goswami, John Mathai, Kameswari K., Lalit Bharadia, Lalit Sankhe, Ajayakumar M.K., Neelam Mohan, Pradeep K. Jena, Rachita Sarangi, Rashmi Shad, Sanjib K. Debbarma, Shyamala J., Simmi K. Ratan, Suman Sarkar, Vijayendra Kumar, Anand P. Dubey, Atul Gupta, Cenita J. Sam, Gowhar Nazir Mufti, Harsh Trivedi, Jimmy Shad, Kaushik Lahiri, Krishnaswamy R., Meera Luthra, Narendra Behera, Padmalatha P., Rajamani G., Rakesh Kumar, Ruchirendu Sarkar, Santosh Kumar A., Subrat Kumar Sahoo, Sunil K. Ghosh, Sushant Mane, Arun Kumar Dash, Bashir Ahmad Charoo, Bikasha Bihary Tripathy, Rajendra Prasad G., Harish Kumar S., Jothilakshmi K., Nihar Ranjan Sarkar, Pavai Arunachalam, Satya Sundar G. Mohapatra, and Saurabh Garge:** Participant recruitment and data collection.

All authors reviewed, provided critical input and approved the final version. The content represents the views of the authors alone and do not necessarily represent the official positions.

## Declaration of Competing Interest

The authors declare that they have no known competing financial interests or personal relationships that could have appeared to influence the work reported in this paper.
