# Supplementary material for: Risk of intussusception after monovalent rotavirus vaccine (Rotavac) in Indian infants: A self-controlled case series analysis
Source: Vaccine. 2021 Jan 3;39(1):78–84. doi: 10.1016/j.vaccine.2020.09.019 (PMC7738754; doi:10.1016/j.vaccine.2020.09.019)
Supplement: Supplementary data 1 [file mmc1.docx]

**Supplementary Documents**

**Index**

| **Sl no** | **Legend** | **Page no** |
| --- | --- | --- |
| 1 | Supplementary Figure SF1: List and locations of the study site institutes in India | 2 |
| 2 | Supplementary Table ST1: The ICD codes for review of the intussusception cases from medical records for quality assurance | 3 |
| 3 | Supplementary Table ST2: The characteristics of the children aged 28-364 days with intussusception selected and not-selected for SCCS analysis | 4 |
| 4 | Supplementary Table ST3: The clinical features among the children aged 28-364 days with intussusception included in the SCCS analysis | 5 |
| 5 | Supplementary Table ST4: The vaccine coverage and median age at vaccination for the children aged 28-364 days with intussusception included in SCCS analysis | 6 |
| 6 | Supplementary Figure SF2: The ages at vaccination and occurrence of intussusception for the children aged 28-364 days who did not receive rotavirus vaccine | 7 |
| 7 | Supplementary Table ST5: The characteristics of the children aged 28-364 days with intussusception who did and didn’t receive rotavirus vaccine included in SCCS analysis | 8 |

Supplementary Figure SF1: List and locations of the study site institutes in India


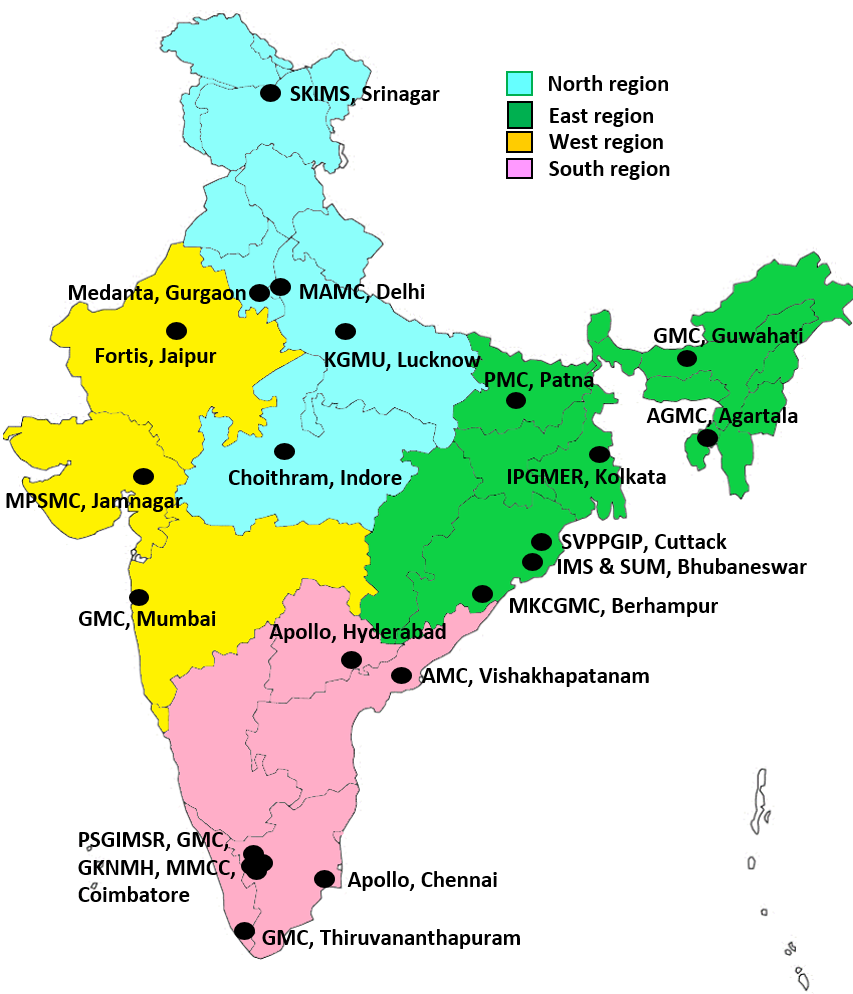


*Note: The map has been prepared for use in the research project. The corresponding author is the creator of the image.*

Supplementary Table ST1: The ICD codes for review of the intussusception cases from medical records for quality assurance

| Clinical conditions considered as suspected cases | Codes | |
| --- | --- | --- |
|  | ICD 10 | ICD 9 |
| Intussusception | K56.1 | 560.0 |
| Volvulus | K56.2 | 560.2 |
| Gallstone ileus | K56.3 | 560.31 |
| Other impaction of intestine | K56.4 | 560.30 |
| Intestinal adhesions with obstruction | K56.5 | 560.81 |
| Other and unspecified intestinal obstruction | K56.6 | 560.9 |
| Ileus, unspecified | K56.7 | 560.1 |
| Paralytic ileus | K56.0 |  |

*ICD: International Classification of Diseases*

Supplementary Table ST2: The characteristics of children aged 28-364 days with intussusception selected and not-selected for SCCS analysis

| Characteristic | Not selected | Selected | Total | p-value |
| --- | --- | --- | --- | --- |
|  | n (%) | n (%) | n (%) |  |
| Gender |  |  |  | 0.450 |
| Boys | 70 (64.2) | 212 (68.2) | 282 (67.1) |  |
| Girls | 39 (35.8) | 99 (31.8) | 138 (32.9) |  |
| Age at intussusception | |  |  | 0.430 |
| ≤12 weeks, n (%) | 5 (4.6) | 6 (1.9) | 11 (2.6) |  |
| 13-24 weeks, n (%) | 29 (26.6) | 91 (29.3) | 120 (28.6) |  |
| 25-36 weeks, n (%) | 45 (41.3) | 137 (44.1) | 182 (43.3) |  |
| 37-52 weeks, n (%) | 30 (27.5) | 77 (24.8) | 107 (25.5) |  |
| Median (IQR) in weeks | 30 (21-36) | 28 (22-35) | 29 (21.5-36) |  |
| Interval between onset and admission | | |  | 0.985 |
| 0-1 days, n (%) | 19 (17.4) | 51 (16.4) | 70 (16.7) |  |
| 2-3 days, n (%) | 41 (37.6) | 119 (38.3) | 160 (38.1) |  |
| 4-5 days, n (%) | 18 (16.5) | 47 (15.1) | 65 (15.5) |  |
| 6-7 days, n (%) | 14 (12.8) | 39 (12.5) | 53 (12.6) |  |
| 8+ days, n (%) | 17 (15.6) | 55 (17.7) | 72 (17.1) |  |
| Median (IQR) in days | 3 (2-6) | 3 (2-6) | 3 (2-6) |  |
| Total, n (%) | 109 (100.0) | 311 (100.0) | 420 (100.0) |  |
| *Note: IQR: Interquartile range (Q1 - left IQR; Q3- right IQR); SCCS: Self-controlled case series;* | | | |  |
| ≤*12 weeks:1-84 days; 13-24 weeks: 85-168 days; 25-36 weeks: 169-252 days; 37-52 weeks: 253-364 days* | | | | |

Supplementary Table ST3: The clinical features among the children aged 28-364 days with intussusception included in the SCCS analysis

| Symptom(s)* | Reported  n (%) | Median duration (IQR) in days |
| --- | --- | --- |
| Vomiting | 245 (78.8) | 1 (1-2) |
| Abdominal pain | 155 (49.8) | 2 (1-3) |
| Excessive crying | 194 (62.4) | 1 (1-2) |
| Abdominal distension | 74 (23.8) | 2 (1-3) |
| Blood in stool | 221 (71.1) | 1 (1-2) |
| Diarrhoea | 77 (24.8) | 2 (1-3) |
| Constipation | 32 (10.3) | 2 (1-3.5) |
| Fever | 77 (24.8) | 2 (1-3) |
| Lethargy | 26 (8.4) | 1 (1-2) |
| Shock | 1 (0.3) | 1 (--) |
| Altered Sensorium | 0 (0) | -- |
| Any symptom | 311 (100) | 2 (1-3) |

*Notes: *Includes multiple symptoms in the same child; IQR: Interquartile range*

Supplementary Table ST4: The vaccine coverage and median age at vaccination for the children aged 28-364 days with intussusception included in SCCS analysis

|  | With RVV | |  | Without RVV | |
| --- | --- | --- | --- | --- | --- |
|  | Number of children  n (%) | Median age (IQR) in weeks |  | Number of children  n (%) | Median age (IQR) in weeks |
| 0 Doses | 259 (83.3) | -- |  | 18 (5.8) | -- |
| Dose-1 only | 4 (1.3) | 7 (6-8) |  | 15 (4.8) | 7 (6-8) |
| Dose-1 and 2 | 5 (1.6) | 11.5 (11-13) |  | 39 (12.5) | 12 (11-13) |
| All 3-doses | 43 (13.8) | 16 (15-18) |  | 239 (76.8) | 16 (15-18) |
| Total | 311 (100) | -- |  | 311 (100) | -- |

*Note: IQR: Interquartile range; RVV: Rotavirus vaccine; SCCS: Self-controlled case series;*

*Oral poliovirus vaccine was used as a proxy for the age at vaccination if RVV not given.*

Supplementary Figure SF2: The ages at vaccination and occurrence of intussusception for the children aged 28-364 days who did not receive rotavirus vaccine


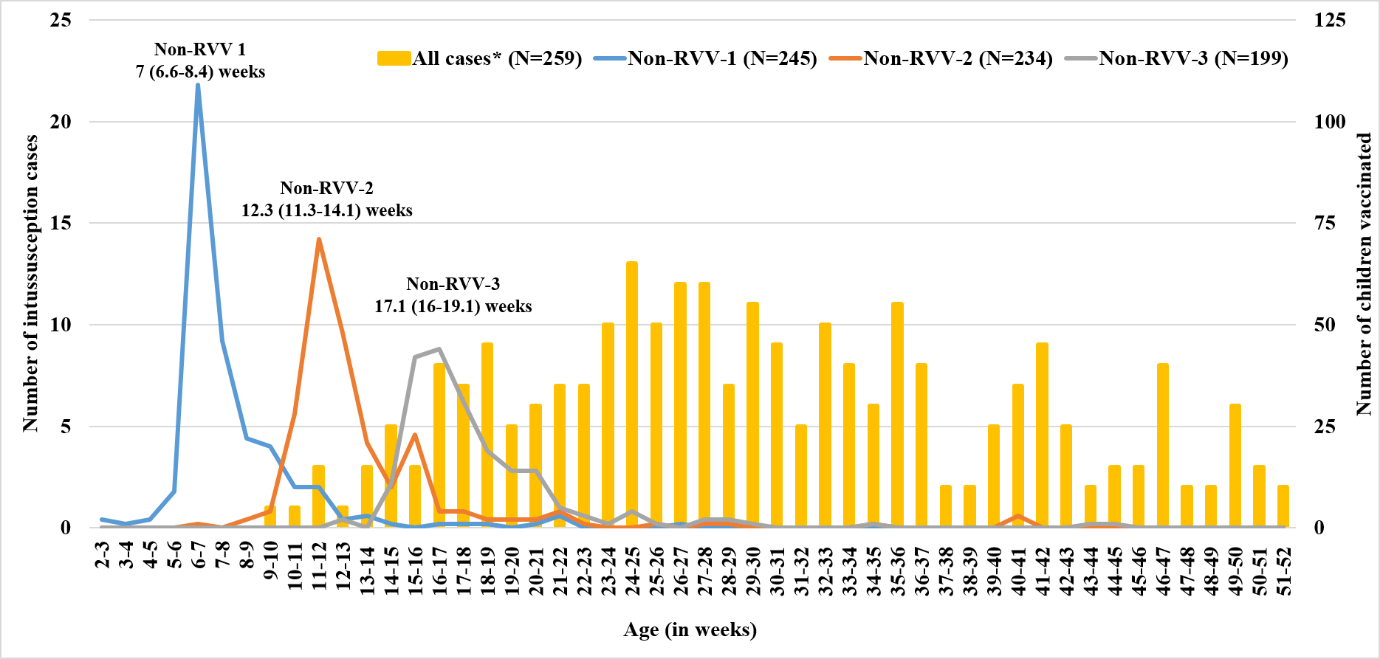


*Note:*Includes the children with intussusception who didn’t receive the rotavirus vaccine (n=259);*

*Ages at vaccination given as median with interquartile range (IQR);*

*Oral poliovirus vaccine was used as a proxy for the age at vaccination if RVV not given.*

Supplementary Table ST5: The characteristics of the children aged 28-364 days with intussusception who did and didn’t receive rotavirus vaccine included in SCCS analysis

|  | | No RVV | RVV | Total | p-value | |
| --- | --- | --- | --- | --- | --- | --- |
| Characteristic | | n (%) | n (%) | n (%) |  |  |
| Gender | |  |  |  | 0.857 | |
| Boys, n (%) | | 176 (68.0) | 36 (69.2) | 212 (68.2) |  | |
| Girls, n (%) | | 83 (32.1) | 16 (30.8) | 99 (31.8) |  | |
| Age at intussusception | |  |  |  | 0.259 | |
| ≤12 weeks, n (%) | | 5 (1.9) | 1 (1.9) | 6 (1.9) |  | |
| 13-24 weeks, n (%) | | 71 (27.4) | 20 (38.5) | 91 (29.3) |  | |
| 25-36 weeks, n (%) | | 114 (44.0) | 23 (44.2) | 137 (44.1) |  | |
| 37-52 weeks, n (%) | | 69 (26.6) | 8 (15.4) | 77 (24.8) |  | |
| Median (IQR) | | 28 (22-36) | 26.5 (19.5-32.5) | 28 (22-35) |  | |
| Interval between onset and admission | | | | | 0.262 | |
| 0-1 days, n (%) | | 48 (18.5) | 3 (5.8) | 51 (16.4) |  | |
| 2-3 days, n (%) | | 97 (37.5) | 22 (42.3) | 119 (38.3) |  | |
| 4-5 days, n (%) | | 38 (14.7) | 9 (17.3) | 47 (15.1) |  | |
| 6-7 days, n (%) | | 31 (12.0) | 8 (15.4) | 39 (12.5) |  | |
| 8+ days, n (%) | | 45 (17.4) | 10 (19.2) | 55 (17.7) |  | |
| Median (IQR) | | 3 (2-6) | 4 (2-7) | 3 (2-6) |  | |
| Total, n (%) | | 259 (100.0) | 52 (100.0) | 311 (100.0) |  | |
| *Note: IQR: Interquartile range (Q1- left IQR; Q3- right IQR); RVV: Rotavirus vaccine; SCCS: Self-controlled case series* | | | | |  |  |
| ≤*12 weeks:1-84 days; 13-24 weeks: 85-168 days; 25-36 weeks: 169-252 days; 37-52 weeks: 253-364 days* | | | | | | |
